# When neglect is neglected: NIHSS observational measure lacks sensitivity in identifying post-stroke unilateral neglect

**DOI:** 10.1136/jnnp-2018-319668

**Published:** 2019-01-23

**Authors:** Margaret Jane Moore, Kathleen Vancleef, Nir Shalev, Masud Husain, Nele Demeyere

**Affiliations:** Department of Experimental Psychology, Radcliffe Observatory Quarter, Cognitive Neuropsychology Centre, University of Oxford, Oxford, UK

**Keywords:** Neuropsychology, Screening, NIHSS, Visual Neglect

## Introduction

Unilateral visual neglect is characterised by lateralised spatial–attentional deficits, resulting in dramatic behavioural impairments.^1^ Neglect negatively impacts functional outcome and needs to be successfully detected in order to inform neglect-specific as well as general post-stroke rehabilitation goals and strategies. It is therefore critically important to evaluate current clinical methods for detecting and measuring the extent of this syndrome.

Observational neurological assessments, such as the National Institutes of Health Stroke Scale (NIHSS), rely predominantly on subjective impression of impairment levels rather than objective measurements.^2^ Although the NIHSS was not designed as an individual diagnostic tool, it is frequently employed as one. However, previous research has suggested that observational assessments may not be sufficiently sensitive to visual neglect.^2–4^ The purpose of this study was to evaluate the diagnostic sensitivity of the NIHSS’ visual neglect item compared with a brief neuropsychological cancellation test and to identify factors which modulate this sensitivity.

## Methods

428 patients who had an acute stroke (mean age, 71 (SD 12.8); mean time post-stroke, 7.3 days (SD 7.4)) completed the NIHSS and Oxford Cognitive Screen (OCS) Cancellation Task (mean interval, 1.2 days). 63.1% of patients completed both tests on the same day and the NIHSS was administered first in 33.9% of cases. The NIHSS Extinction/Inattention and Visual Field items were considered in this investigation, with Extinction/Inattention scores of 0 (none), 1 (mild) or 2 (profound) and Visual Field scores of 0 (normal), 1 (partial) or 2 (complete).

The OCS is a brief stroke-specific cognitive screen which includes a highly sensitive Cancellation Task.^5^ This test was therefore used as the comparison standard for NIHSS sensitivity calculations. In this Cancellation Task, patients are instructed to search for and mark complete heart outlines while ignoring incomplete hearts. Egocentric neglect was scored by subtracting the number of targets identified on the left and right side of the page while allocentric neglect was scored by subtracting the number of right-gap and left-gap hearts identified. Egocentric asymmetries larger than 3 and allocentric asymmetries greater than 1 represent significant impairment.^5^

## Results

First, the sensitivity of the NIHSS to neglect was evaluated. 83/428 (19.4%) and 199/428 (46.5%) patients exhibited neglect as reported by the NIHSS and OCS, respectively. In comparison with the OCS, the NIHSS exhibited a high neglect specificity (91.2%), though a low sensitivity (31.6%). Interestingly, the Extinction/Inattention Item was not found to be significantly more sensitive to neglect than the Visual Field Item (sensitivity, 28.1%; McNemar’s, χ^2^=0.735, p=0.39).

**Figure 1:**
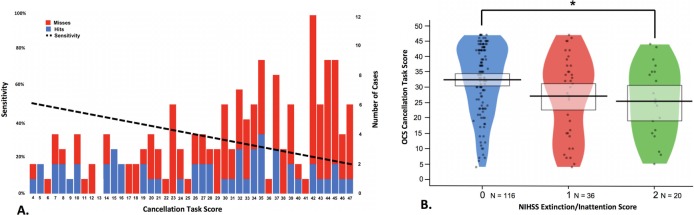
(A) Illustration of the relationship between cancellation score and National Institutes of Health Stroke Scale (NIHSS) sensitivity. The number of neglect cases of each severity level is denoted by bar height (right y-axis). Cases which were successfully identified by the NIHSS are blue and missed cases are red. The sensitivity linear regression line is plotted as the dashed line. (B) Relationship between NIHSS Inattention/Extinction Score and Oxford Cognitive Screen (OCS) Cancellation Task score egocentric neglect patients. Score distribution is represented by plot width, points represent individual cases, and rectangles represent upper and lower quartiles. Lower cancellation scores represent more severe neglect. *p<0.05.

Next, the relationship between NIHSS sensitivity and neglect severity was investigated (figure 1). A regression analysis demonstrated that patients with milder neglect on the OCS cancellation were significantly less likely to be identified by the NIHSS than patients with more severe neglect (R^2^=0.107, F(1,42)=5.016, p=0.030, β=−0.327). Similarly, there was a significant difference in cancellation total for neglect patients with different Extinction/Inattention Item scores (F(2,169)=4.777, p=0.010). However, there was large individual variability in all NIHSS item severity categories and the NIHSS was still found to have a low sensitivity (38.1%) when only the most severe neglect patients (cancellation totals <10/50) were considered.

Finally, the relationship between neglect subtypes and NIHSS sensitivity was investigated. The NIHSS was found to be more likely to detect left-lateralised (sensitivity, 58.3%, N=111) than right-lateralised neglect (sensitivity, 18.7%, N=75). This substantial difference in sensitivity occurred despite the fact that there was no significant difference in the severity of cases of right and left neglect in this sample; t(157)=1.767, p=0.079. Finally, the NIHSS was found to be more sensitive to co-occurring egocentric and allocentric neglect (sensitivity, 68.8%, N=54) than pure egocentric (28.8%, N=118) or allocentric (25.9%, N=27) neglect cases.

## Discussion

The NIHSS was found to have poor sensitivity compared with the Cancellation Task. While the NIHSS was more likely to successfully identify severe versus mild cases of neglect, it frequently failed to detect even the most severe cases, demonstrated by the large spread of the neglect severity data in each NIHSS inattention item scoring category. Visual Field Item sensitivity to neglect was not found to differ from that of the Extinction/Inattention Item suggesting that the frequent failure of clinical observation alone to detect neglect may also be associated with a misattribution of neglect impairments to visual field deficits (eg, hemianopia). Additionally, the NIHSS did not reliably detect less common subtypes of visual neglect, such as right-sided and allocentric neglect. These results indicate that reliable detection of post-stroke neglect requires, at the minimum, the inclusion of a simple cancellation task.

These findings have important implications when considered in the context of post-stroke care. First, relying solely on observational assessments like the NIHSS will lead to an underestimation of neglect prevalence. Failure to detect neglect might alter perceived clinical prognosis or lead to a lack of necessary care packages being put into place. Second, the failure of clinical observation to detect neglect may be associated with a general misunderstanding of visual neglect. This investigation demonstrates that observational assessments are more likely to detect leftward than rightward neglect, regardless of the severity of these deficits. This could potentially be explained by expectation bias, as visual neglect is commonly mischaracterised as an exclusively leftward deficit. Finally, with regards to the heterogeneity of neglect, observational assessment was shown to be more sensitive to the more broadly understood egocentric than to allocentric neglect. While the fractionation of neglect may appear only theoretically relevant, previous research has contradicted this by demonstrating that allocentric neglect is associated with poorer long-term functional outcomes than egocentric neglect.^4^ A portion of this egocentric/allocentric sensitivity difference might result from a lack of clinician awareness of the fractionation of neglect. NIHSS training protocols could be improved to promote a more thorough understanding of neglect as a syndrome and to include a simple neuropsychological assessment to help detect less common neglect subtypes.

In conclusion, the inclusion of an objective, brief standard cancellation task in post-stroke screening is necessary to reliably identify visual neglect, differentiate neglect from visual field impairments and eliminate subjective biases.
